# Genome-Wide Identification of *LeBAHDs* in *Lithospermum erythrorhizon* and In Vivo Transgenic Studies Confirm the Critical Roles of *LeBAHD1/LeSAT1* in the Conversion of Shikonin to Acetylshikonin

**DOI:** 10.3390/life12111775

**Published:** 2022-11-03

**Authors:** Xuan Wang, Zhuoyu He, Huan Yang, Cong He, Changyi Wang, Aliya Fazal, Xiaohui Lai, Liangjie Yang, Zhongling Wen, Minkai Yang, Shenglin Ma, Wencai Jie, Jinfeng Cai, Tongming Yin, Bao Liu, Yonghua Yang, Jinliang Qi

**Affiliations:** 1 State Key Laboratory of Pharmaceutical Biotechnology, Institute for Plant Molecular Biology, School of Life Sciences, Nanjing University, Nanjing 210023, China; 2 Co-Innovation Center for Sustainable Forestry in Southern China, Nanjing Forestry University, Nanjing 210037, China; 3 Yili Key Laboratory of Applied Research and Development on Active Ingredients of Chinese Herbal Medicine, Yili National Agricultural Science and Technology Park at Xinjiang, Yili 835600, China; 4 Key Laboratory of Molecular Epigenetics of the Ministry of Education (MOE), Northeast Normal University, Changchun 130024, China

**Keywords:** *Lithospermum erythrorhizon*, acetylshikonin, shikonin, BAHD, LeSAT1, hairy root

## Abstract

The BAHD acyltransferase family is a unique class of plant proteins that acylates plant metabolites and participates in plant secondary metabolic processes. However, the BAHD members in *Lithospermum erythrorhizon* remain unknown and uncharacterized. Although the heterologously expressed *L. erythrorhizon* BAHD family member LeSAT1 in *Escherichia coli* has been shown to catalyze the conversion of shikonin to acetylshikonin in vitro, its in vivo role remains unknown. In this study, the characterization, evolution, expression patterns, and gene function of *LeBAHDs* in *L. erythrorhizon* were explored by bioinformatics and transgenic analysis. We totally identified 73 *LeBAHDs* in the reference genome of *L. erythrorhizon*. All *LeBAHDs* were phylogenetically classified into five clades likely to perform different functions, and were mainly expanded by dispersed and WGD/segmental duplication. The in vivo functional investigation of the key member *LeBAHD1/LeSAT1* revealed that overexpression of *LeBAHD1* in hairy roots significantly increased the content of acetylshikonin as well as the conversion rate of shikonin to acetylshikonin, whereas the CRISPR/Cas9-based knockout of *LeBAHD1* in hairy roots displayed the opposite trend. Our results not only confirm the in vivo function of *LeBAHD1/LeSAT1* in the biosynthesis of acetylshikonin, but also provide new insights for the biosynthetic pathway of shikonin and its derivatives.

## 1. Introduction

Red naphthoquinones are the main secondary metabolites accumulated in the roots of a few *Boraginaceae* plants such as *Lithospermum erythrorhizon* and *Arnebia euchroma*, among which shikonin and its derivatives such as acetylshikonin, isobutyrylshikonin, β,β-dimethylacrylshikonin have been shown to have broad medicinal value and industrial applications [1,2,3]. Acetylshikonin is the main naphthoquinone in the root periderm of the traditional Chinese medicine plant *L. erythrorhizon*, accounting for about 50% of the total [4]. Acetylshikonin has attracted much attention as a potential anticancer drug because of its ability to inhibit cell proliferation [5] and induce cell autophagy [6], and can inhibit the growth of colorectal cancer [7], liver cancer [8], oral cancer [9], melanoma [10], etc., and is less toxic to normal human cells [11,12]. Therefore, it has become urgent and crucial to dissect the biosynthetic mechanism of acetylshikonin and improve its yield in plant-derived materials.

A two stage culture system, where: (1) cell cultures [13] or hairy roots [14] of *L. erythrorhizon* are cultured in a growth medium (B5 medium) under light for proliferation; (2) the proliferated hairy roots or cells are transferred to the production medium (M9 medium) in darkness to induce the production of shikonin and its derivatives, has been successfully developed to study the biochemical and molecular mechanisms of the biosynthesis of shikonin and its derivatives, as well as the yield improvement of these useful compounds [15]. Based on this culture system, many key genes, such as *LePGT* [16], *LeC4H* [17], *LePAL* [18], *Le4CL* [19], hydroxylase *CYP76B74* [20], *CYP76B101* [21] *LeDSH* [22], etc., have been excavated, and the pathway for shikonin biosynthesis was well characterized. However, the key enzymes that catalyze the conversion of shikonin to its derivatives have not been fully elucidated.

Most shikonin derivatives are produced by the acylation of different types of acyl groups from CoA thioester acyl donors to shikonin side chains catalyzed by acyltransferases [23]. The plant BAHD acyltransferases family, which was named after the initials of the four characterized members (benzylalcohol O-acetyltransferase from *Clarkia breweri*, BEAT; anthocyanin O-hydroxycinnamoyltransferases from *Petunia*, *Senecio*, *Gentiana*, *Perilla*, and *Lavandula*, AHCTs; anthranilate N-hydroxycinnamoyl/benzoyltransferase from *Dianthus caryophyllus*, HCBT; deacetylvindoline 4-O-acetyltransferase from *Catharanthus roseus*, DAT) [24,25], has been shown to catalyze the transfer of acyl moieties to a variety of acceptor molecules and were involved in the biosynthesis of various natural secondary metabolites such as flavonoids, phenols, alkaloids, anthocyanins, and volatile esters [25,26]. Recently, a shikonin O-acyltransferases gene (*LeSAT1*) belonging to the BAHD gene family was identified in *L. erythrorhizon*, its function of catalyzing acetyl-CoA, isobutyryl-CoA, and isovaleryl-CoA as acyl donors to generate acetylshikonin, isobutyrylshikonin, and isovalerylshikonin was verified by in vitro enzyme activity, and the acetylation activity of *LeSAT1* was the strongest [27]. However, the in vitro catalytic experiments of *LeSAT1′s* functions need to be further confirmed by in vivo transgenic studies. After that, Tang [28] conducted a preliminary screening and evolutionary process exploration of the alkannin/shikonin O-acyltransferase gene (i.e., AAT/SAT)-like superfamily members in *L. erythrorhizon* with AAT/SAT’s amino acid sequences (i.e., BBV14785.1 and BBV14786.1) as the queries. However, the genome-wide identification and characterization of the *LeBAHD* gene family in *L. erythrorhizon* still remains elusive. 

Here, we conducted genome-wide identification of *LeBAHD* members in *L. erythrorhizon*, fully characterized their regulatory elements and expression patterns, and predicted gene functions based on their evolutionary status; moreover, we confirmed the in vivo effect of LeBAHD1/LeSAT1 in catalyzing the conversion of shikonin to acetylshikonin in plants using overexpression and CRISPR/Cas9 strategies based on the two-stage culture system of *L. erythrorhizon* transgenic hairy roots. Our study not only pioneers the use of the CRISPR/Cas9 gene knockout system on the hairy roots of *L. erythrorhizon*, laying a practical foundation for more precisely revealing the biochemical and molecular mechanisms underlying the biosynthesis and regulation of shikonin and its derivatives, but also provides a method for the efficient biosynthesis of acetylshikonin through genetic engineering of *L. erythrorhizon*.

## 2. Materials and Methods

### 2.1. Plant Materials and Growth Conditions

The seeds of *Lithospermum erythrorhizon* Sieb. et Zucc collected in Tuoyaozi Town, Huanan County, Jiamusi City, Heilongjiang Province, China (130°78′81″ E/46°23′95″ N), were dispersed in wet sand for about one month at 4 °C in the dark. After germination, the seeds were transplanted into the soil in the greenhouse for cultivation at 25 °C with a 16 h day/8 h night photoperiod and 100 μmol m^−2^ s^−1^ light intensity until the seedlings developed 8 leaves, which were used as explants for hairy root induction.

### 2.2. Identification of LeBAHD Superfamily

To identify the putative *LeBAHD* genes from *L. erythrorhizon*, several approaches were synergistically employed. The BAHD family characteristic domain (Pfam domain: PF02458) and HMMER 3.0 were used to search the genome of *L. erythrorhizon* for candidate *LeBAHD* genes with E-values less than e^−10^ [29]; in addition, 55 AtBAHD protein sequences of *Arabidopsis thaliana* were used to blastp the *L. erythrorhizon* genome; then, redundant sequences and abnormal sequences (including incomplete PF02458 domain, lacking initiation codon and/or termination codon, and lack of one or two characteristic motifs: HXXXD and DFGWG) identified by Batch CD-Search (https://www.ncbi.nlm.nih.gov/Structure/bwrpsb/bwrpsb.cgi, accessed on 10 September 2022) and Motif analysis (https://meme-suite.org/meme/tools/meme, accessed on 10 September 2022) were then removed.

### 2.3. Bioinformatic Analysis

The ExPASy website (https://web.expasy.org/protparam/, accessed on 10 September 2022) was used to analyze the chemical characteristics of LeBAHDs, and the subcellular localization prediction was carried out using Cell-PLoc 2.0 (http://www.csbio.sjtu.edu.cn/bioinf/Cell-PLoc-2/, accessed on 10 September 2022). The full-length amino acid sequence of LeBAHDs was subjected to a conserved motif analysis using the MEME program (https://meme-suite.org/meme/tools/meme, accessed on 10 September 2022) with the default setting of 15 motifs. The gene structure was analyzed using the Gene Structure Display Server 2.0 (http://gsds.gao-lab.org/, accessed on 10 September 2022). Gene duplication types of *LeBAHDs* were analyzed using the ‘MCScanX’ and ‘duplicate_gene_classifier’ programs implemented in the MCScanX package. The cis-acting elements of the promoter region (2000 bp sequence upstream of the start codon) of the *LeBAHD* genes were analyzed using the PlantCARE program (http://bioinformatics.psb.ugent.be/webtools/plantcare/html/, accessed on 10 September 2022).

The phylogenetic trees were constructed using the following three steps: (1) the amino acid sequences of LeBAHD family members were aligned via prank v17042751 (key parameter: –F–codon); (2) the preliminary alignment was trimmed using trimAL v.1.2.rev59 (key parameter: –gt 0.50); (3) the trimmed alignment was then used to construct the phylogenetic tree via iqtree-2.2.0-Windows according to the Maximum Likelihood (ML) method with 1000 bootstrap replications. The constructed phylogenetic tree was embellished using iTOL (https://itol.embl.de/, accessed on 10 September 2022). Protein sequences of the published BAHD family members (Table S1) used for LeBAHDs phylogenetic analysis were retrieved from the Genbank database.

To explore the specific expression patterns of *LeBAHDs* in different *L. erythrorhizon* tissues, RNA-seq data from *L. erythrorhizon* mature roots (MR), root periderm (PD), root cortex (CT), root stele (SE), mature leaves + stems (ML) and flowers (FL) were downloaded from the NCBI SRA (accession ID: SRP141330) and used for gene expression analysis. The heatmap was drawn in Rstudio using pheatmap. 

### 2.4. cDNA Cloning of LeBAHD1/SAT1, Plasmid Construction and Transformation

Total RNA of the red roots of *L. erythrorhizon* seedlings was extracted using the FastPure Plant Total RNA Isolation Kit (Vazyme, #RC401, Nanjing, China) and cDNA was synthesized by reverse transcription with the HiScript III 1st Strand cDNA Synthesis Kit (+gDNA wiper) (Vazyme, #R312, Nanjing, China). With the specific primers (Table S2) designed by Primer Premier 5.0, the full-length open-reading frame (ORF) of *LeBAHD1*/*LeSAT1* (1317 bp, GenBank number: LC520137.1) was amplified with a high-fidelity enzyme (Vazyme, #P515, Nanjing, China) using the above cDNA as the template. The PCR parameters were as follows: 95 °C for 3 min, followed by 34 cycles at 95 °C for 15 s, 58 °C for 15 s, and 72 °C for 1 min, with a final extension at 72 °C for 10 min.

For the construction of *LeBAHD1*/*LeSAT1* overexpression vector (pBI121*–LeBAHD1*/*LeSAT1–eGFP*), the above amplified full-length ORF of *LeSAT1* was subcloned into the *Xba* I/*Bam* HI sites of the plant expression vector pBI121–eGFP by homologous recombination. The *LeBAHD1*/*LeSAT1* knockout vector (pYLCRISPR/Cas9–*LeBAHD1*/*LeSAT1* was constructed using the CRISPR/Cas9 vector system (pYLCRISPR/Cas9P_ubi_-H, sgRNA promoter of *A. thaliana* AtU6-29). The sgRNA target was designed on the sense and antisense strand of its first exon according to the *LeSAT1* gene sequence, and the target sequence was imported into the sgRNA expression box by overlapping PCR. The PCR parameters were as follows: 95 °C for 3 min, followed by 30 cycles at 95 °C for 15 s, 58 °C for 15 s, and 68 °C for 20 s, with a final extension at 68 °C for 2 min. Then, the sgRNA expression box was added to the skeleton vector through the digestion at *Bsa* I site.

All the recombined expression vectors were respectively transformed into competent cells of *Escherichia coli* strain Top10 by heat shock and verified before being introduced into competent cells of *Agrobacterium rhizogenes* strain ATCC15834 by electroporation. All the primers used for the vector construction, as well as the verification and identification of *A. rhizogenes* strain ATCC15834 harboring the constructs, are listed in Table S2.

### 2.5. Hairy Root Induction, Culture, and Validation

For transgenic hairy root induction and culture, the explants cut from seedling leaves (1.0–1.5 cm) were placed in MS cultures containing 0.2 mg/L 6-benzylaminopurine and 2 mg/L 2,4-dichlorophenoxyacetic acid and incubated in the dark at 25 °C for 2 days. Meanwhile, the strain ATCC15834 was successfully transformed with pBI121–eGFP (EV), pBI121–*LeBAHD1*/*LeSAT1* (OE), pYLCRISPR/Cas9Pubi-H (MH), or pYLCRISPR/Cas9–*LeBAHD1*/*LeSAT1* (MH-K) plasmid, and incubated in liquid YEB medium with 50 mg/L kanamycin on a rotary shaker at 120 rpm at 26–28 °C to an OD_600_ of 0.6. Then acetosyringone was added to the above bacterial culture medium to a final concentration of 0.1 mM to prepare an infection solution. The precultured leaf explants were placed in the infection solution and treated in the dark at 28 °C for 30 min. Then, the explants were incubated in MS solid medium at 26 °C for 2 days in the dark. Afterwards, the infected explants were washed three times with sterilized water, and then the explants were put into MS solid medium supplemented with 500 mg of cefotaxime and cultured at 26 °C in the dark. Hairy roots appeared after 2–3 weeks of cultivation. The developed hairy roots of approximately 2 cm in length were excised from the infection sites and then subcultured on solid 1/2 B5 medium supplemented with 500 mg/L cefotaxime at 26–28 °C for about 1 week to eliminate *Agrobacterium*. The concentration of cefotaxime was constantly reduced to eliminate *Agrobacterium* completely. Finally, the hairy roots of EV, MH, OE or MH-K were transferred into antibiotic-free and hormone-free 1/2 B5 solid medium for continuous growth at 26–28 °C. Then, the *rolC* of *A. rhizogenes* or hygromycin resistance gene (*HPT*) gene was amplified in the genomic DNA extracted from transgenic hairy roots to confirm that the hairy roots were transgenic hairy roots, not aerial roots. The PCR parameters were as follows: 95 °C for 3 min, followed by 34 cycles at 95 °C for 15 s, 54 °C for 15 s, and 72 °C for 45 s, with a final extension at 72 °C for 10 min. The transgenic efficiency of the OE hairy roots was verified by quantitative real-time PCR (RT-qPCR) using gene specific primers. The RT-qPCR parameters were as follows: 95 °C for 5 min, followed by 40 cycles at 95 °C for 10 s and 60 °C for 30 s, with a final extension at amplification conditions of a default dissolution curve. Primers were designed on 200–300 bp sequences on both sides of the sgRNA target, and the target sequence was amplified from genomic DNA extracted from MH-K hairy roots to detect its gene editing effect; the PCR parameters were as follows: 95 °C for 3 min, followed by 34 cycles at 95 °C for 15 s, 56 °C for 15 s, and 72 °C for 40 s, with a final extension at 72 °C for 10 min. All primers used for transgenic hairy root validation are listed in Table S2.

The subcultured hairy roots were transferred into a 50 mL Erlenmeyer flask containing 20 mL of 1/2 B5 liquid medium at 28 °C under light with shaking at 100 rpm for rapid proliferation. Then, these proliferated hairy roots were transferred from 1/2 B5 proliferation medium into 20 mL of M9 production medium and incubated in the dark at 28 °C and 100 rpm to induce the production of shikonin and its derivatives, as described in a previous report [30].

### 2.6. eGFP Fluorescence Detection of Hairy Roots

The transgenic hairy roots expressing 35S: eGFP or 35S: *LeBAHD1*/*LeSAT1*–eGFP were directly put on a confocal laser scanning fluorescence microscope (LSFM, FV10-ASW, Olympus, Japan) for the observation of eGFP fluorescence. The fluorescence signal of GFP was excited at 488 nm and the emission wavelength was detected at 510 nm as previously reported [31].

### 2.7. RNA Extraction and RT-qPCR Analysis

Total RNA was extracted from different transgenic hairy root lines (EV, OE, MH, or MH-K), or from different tissues of *L. erythrorhizon* (root, stem, or leaf) using the FastPure Plant Total RNA Isolation Kit (Vazyme, #RC401, Nanjing, China). The RNA purity and integrity were assessed based on the A_260_/A_280_ absorbance ratio and 1.0% agarose gel electrophoresis. Approximately 1 µg of total RNA was used to synthesize cDNA by reverse transcription with the HiScript III 1st Strand cDNA Synthesis Kit (+gDNA wiper) (Vazyme, #R312, Nanjing, China), and RT-qPCR was performed using ChamQ Universal SYBR qPCR Master Mix (Vazyme, #Q711, Nanjing, China) with gene-specific primers (Table S2) on an Applied Biosystems 7500 Real-Time PCR System and StepOnePlus™ Real-Time PCR System. The RT-qPCR parameters were as follows: 95 °C for 5 min, followed by 40 cycles at 95 °C for 10 s and 60 °C for 30 s, with a final extension at amplification conditions of a default dissolution curve. Gene expression levels of each sample were normalized relative to the glyceraldehyde-3-phosphate dehydrogenase encoding gene (*GAPDH*) mRNA as an internal standard, and were calculated using the 2^−ΔΔCt^ method [32]. At least three independent experiments were performed for each analysis.

### 2.8. Extraction and Quantitative Determination of Shikonin and Acetylshikonin

Shikonin and its derivatives were extracted and quantified from hairy roots and the M9 medium as previously reported [30]. In brief, both fresh hairy roots (approximately 0.5 g) and the M9 production medium (20 mL) were extracted with petroleum ether and then dissolved in 1 mL of methanol following rotary evaporation. Using shikonin and its derivatives, including shikonin and acetylshikonin, as standards, a total of 2 µL of methanolic extract was analyzed using high performance liquid chromatography (HPLC) [1].

### 2.9. Statistical Analysis

All data are presented as means with standard deviations (SDs). The means ± SD values were calculated for each material using Microsoft Excel 2019. Statistical analysis using the Student’s *t*-test was performed using GraphPad Prism 8. * *p* < 0.05, ** *p* < 0.01, *** *p* < 0.001, **** *p* < 0.0001.

## 3. Results

### 3.1. Identification and Characterization of LeBAHD Genes in L. erythrorhizon Genome

In order to have a comprehensive understanding of the characteristics and functions of BAHD family genes in *L. erythrorhizon*, 104 members were initially screened out from the *L. erythrorhizon* genome based on sequences homology and the conserved BAHD superfamily feature domain (Pfam: PF02458). A total of 73 genes were then identified following a series of de-redundant and abnormal sequence exclusions (Tables S3 and S4). Analysis of the basic physicochemical properties of putative LeBAHDs showed that the full length of the coding region is between 1068 and 1464 bp, the molecular weight of LeBAHD proteins is about 39.3–54.17 kDa, 47.9% of LeBAHDs were unstable proteins, and the theoretical isoelectric points (pI) of proteins varied widely from 4.86 to 9. In addition, subcellular localization predictions suggested all the proteins likely function in the cytoplasm. The relevant information of the putative LeBAHDs is listed in Table S4.

**Figure 1:**
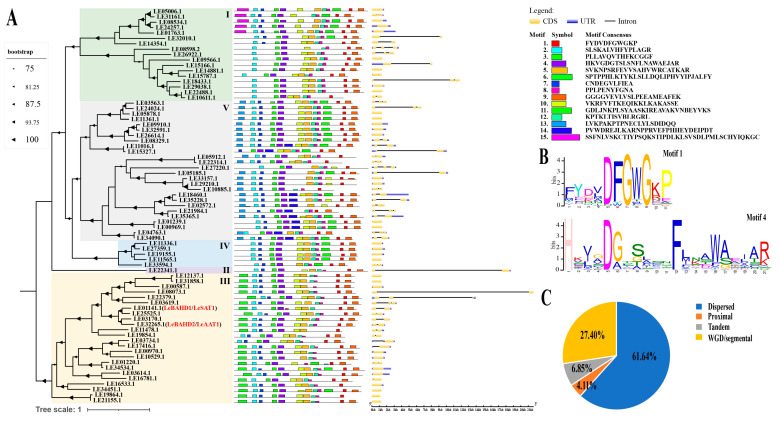
Phylogenetic relationships, conserved motifs, gene structures, and the expansion events of *LeBAHDs*. (**A**) The phylogenetic tree, conserved motifs, and gene structures of 73 *LeBAHDs* identified in *L. erythrorhizon*. I–V represent different clades in the phylogenetic tree. (**B**) The logos of Motif 1 and Motif 4 of LeBAHD proteins were identified using the MEME search tool, and Motif 1 and Motif 4 have conserved domains of DFGWG and HXXXD, respectively. (**C**) Gene duplication types and their proportions in 73 *LeBAHDs*.

The distribution of protein motifs may provide insight into the functional diversity of a gene family’s members. MEME detected fifteen motifs in the predicted full-length protein sequences of LeBAHDs (Figure 1A and Figure S1), and all LeBAHDs contained both motif 1 (FYDVDFGWGKP) and motif 4 (HKVGDGTSLSNFLNAWAEJAR), which correspond to the conserved domains DFGWG and HXXXD, respectively, associated with enzymatic activity. Moreover, the HXXXD domain is conserved in all potential genes, whereas the DFGWG domain demonstrated heterogeneity (Figure 1B). Consistent with the classification of LeBAHDs into five clades in their phylogenetic tree, the type and distribution of conserved motifs within the same subclade are identical (Figure 1A). Gene structure analysis using the GSDS tool revealed that the number of exons in *LeBAHDs* ranged from one to six, and that 31 proteins lacked introns (Figure 1A).

To better comprehend the evolutionary history of LeBAHDs, we analyzed and counted the duplication type of all *LeBAHD*s. The results showed that more than half of *LeBAHDs* were derived from dispersed duplication, 27.39% of the members may have evolved through WGD/segmental, and the remaining genes were generated from tandem and proximal duplications (Figure 1C, Table S4).

### 3.2. Phylogenetic Classes and Function Analysis of LeBAHD Proteins

**Figure 2:**
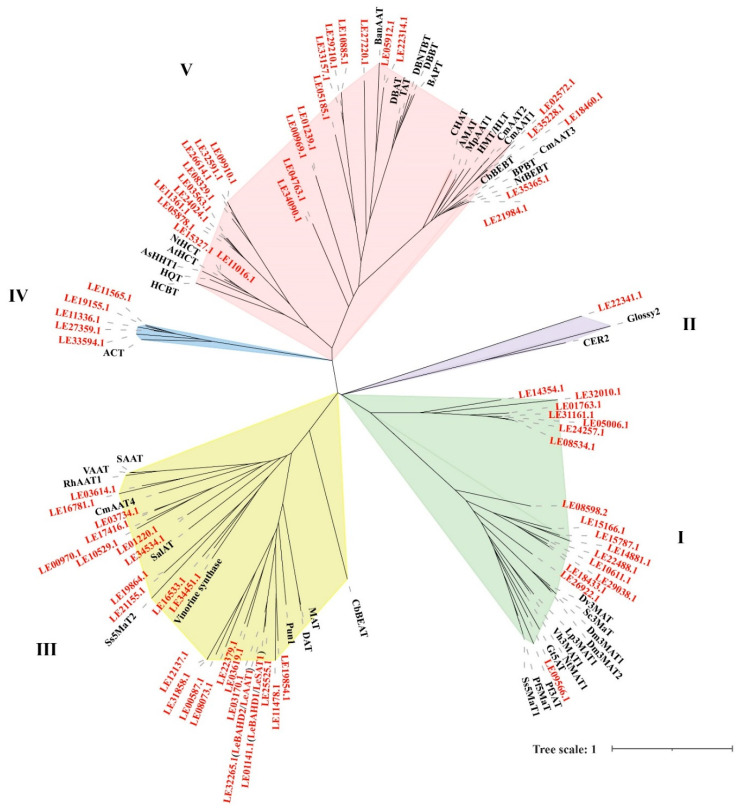
The phylogenetic tree of 119 BAHDs from 28 species including 73 LeBAHDs identified from *L. erythrorhizon* and 46 BAHD used in D’Auria’s study [27]. The phylogenetic tree was constructed based on full-length protein sequences using the maximum likelihood method with 1000 replicates. I–V represent different clades in the phylogenetic tree.

Similar evolutionary constraints apply to proteins with similar functions [33]. To further speculate on the evolutionary classification and functions of LeBAHDs, we constructed a phylogenetic tree of 119 BAHD amino acid sequences using the maximum likelihood method (Figure 2), including 73 LeBAHDs and 46 canonical sequences of BAHDs (Tables S1 and S4). LeBAHDs were divided into five clades, which was in accordance with the evolutionary relationships described by D’Auria [26]. Based on the phylogenetic tree, 17 LeBAHDs belonging to clade I are closely related to Dv3MAT in *Dahlia variabilis*, Gt5AT in *Gentiana triflora*; LE22341.1, closely related to Glossy2 in maize and CER2 in *A. thaliana* is the only one member classified in clade II. There are 24 LeBAHDs in clade III, which are closely related to proteins with acetylation function, such as Vinorine synthase identified in *Rauvolfia serpentina* and DAT identified in *C. roseus*; LeBAHD1/LeSAT1 is closely related to Le25525.1, and they converge with LeAAT1 and LE03170.1 to form a small branch in clade III. The five LeBAHDs in clade IV are closest to ACT in *Hordeum vulgare*; clade V contains the highest number of LeBAHDs, which are closely related to various proteins with different functions, such as BanAAT in *Musa sapientum* and NtBEBT in *Nicotiana tabacum*.

### 3.3. Expression Patterns of LeBAHD Genes in Different Tissues of L. erythrorhizon

**Figure 3:**
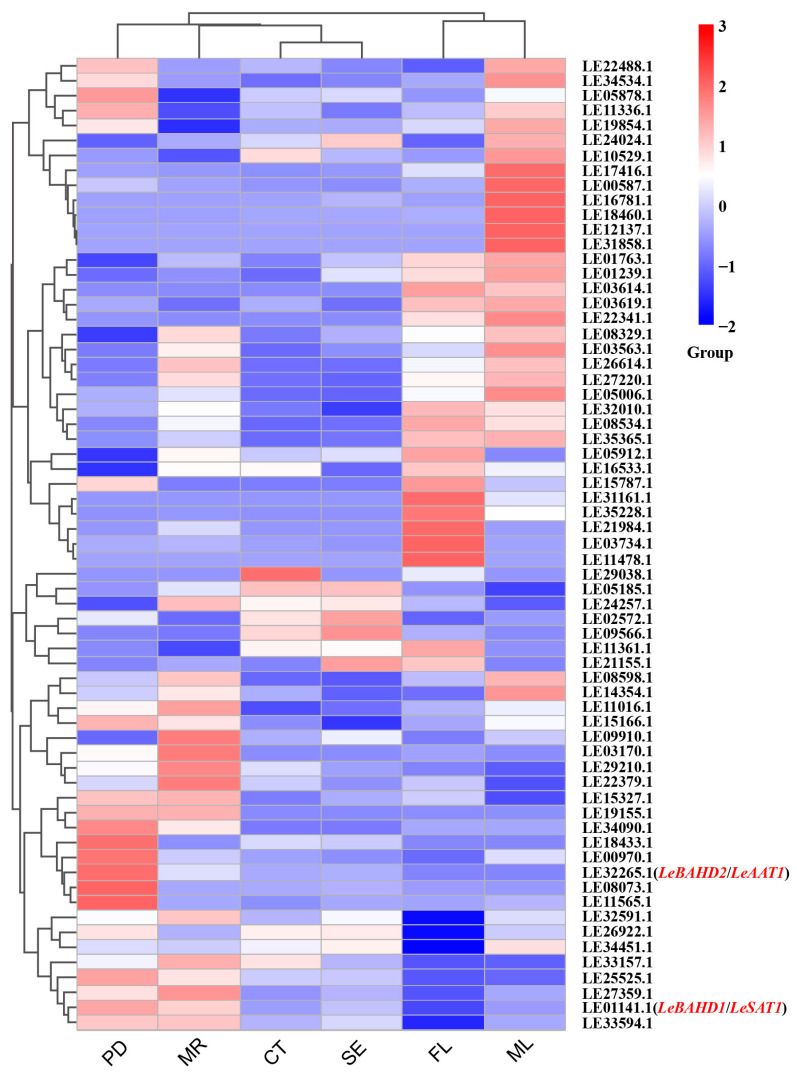
Expression patterns of *LeBAHDs* in different tissues of *L. erythrorhizon* seedlings based on transcriptome data. MR: mature roots; PD: root periderm; FL: flowers; ML: mature leaves + stems; CT: root cortex; SE: root stele.

To further explore the functions of LeBAHD members in *L. erythrorhizon*, we analyzed the expression patterns of 73 *LeBAHDs* in different tissues of *L. erythrorhizon* (ML: mature stems + leaves; FL: mature flower; PD: root periderm; MR: mature root; CT: root cortex; SE: root stele) using the published transcriptome data (NCBI SRA accession ID: SRP141330). The heat map results showed that the expression of *LeBAHDs* was distributed in almost every tissue (Figure 3). Among the expressed genes, the members LE17416.1, LE00587.1, LE16781.1, LE12137.1, and LE31858.1 in clade III and LE18460.1 in clade V had significantly higher expression levels in leaves than in other tissues; 12 *LeBAHD* genes showed high transcription levels in flowers, including LE03614.1, LE05912.1 and LE11478.1; LE29038.1, LE09566.1, LE02572.1 and LE21155.1 were preferentially expressed in root cortices or root steles; Importantly, some *LeBAHDs*, especially *LeBAHD1*/*LeSAT1* and LE25525.1, showed elevated transcription levels in mature root and/or root periderm where shikonin and its derivatives are biosynthesized (Figure 3).

In order to verify the root specific expression pattern of *LeBAHD1*/*LeSAT1*, the relative expression levels of this gene in roots, stems and leaves of *L. erythrorhizon* seedlings were determined by RT-qPCR. Consistent with the transcriptome data, the results show that *LeBAHD1*/*LeSAT1* is preferentially expressed in the roots, showing that the expression level in roots was 738 times and 119 times higher than that in leaves and stems, respectively (Figure S2).

### 3.4. Analysis of Cis-Acting Elements in the Promoters of LeBAHD Genes

**Figure 4:**
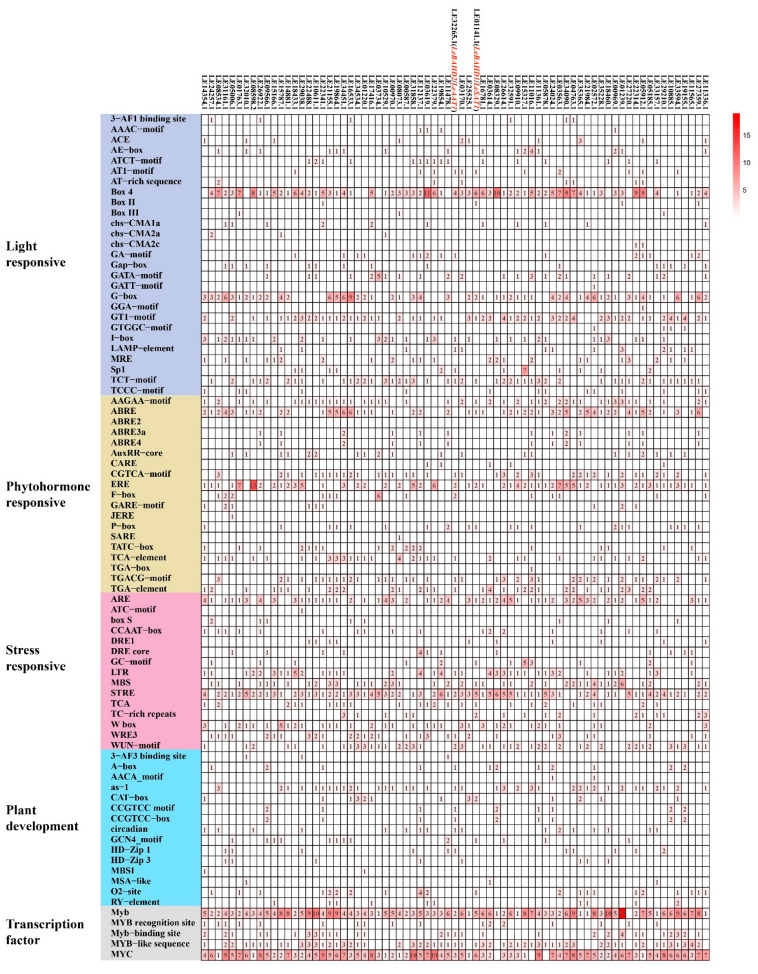
Analysis of cis-acting elements in *LeBAHDs*’ promoters. Different colors and numbers in the grids indicate the numbers of different types of cis-acting element types in the promoters.

To assess putative cis-acting elements in the promoter regions of *LeBAHDs* that regulate their expression, the PlantCARE tool was used to investigate the 2000 bp sequence upstream of the start codon of 73 *LeBAHD* genes. In all *LeBAHDs* promoters, 12,138 cis-acting elements of 102 types were predicted (Table S5). The most significant environmental regulator of *LeBAHDs* among them was light signals, followed by phytohormones (Figure 4 and Figure S3). A total of 27 types of light-responsive elements were identified in the *LeBAHD* promoter sequences, including Box 4, GA-motif, G-box, TCT-motif, etc. (Figure 4). Meanwhile, 19 different types of phytohormone-related cis-elements were identified, with the majority of *LeBAHD* promoters containing phytohormone-responsive binding sites: abscisic acid-responsive elements (ABRE motif and AAGAA-motif), MeJA-responsive elements (CGTCA-motif and TGACG-motif), ethylene-responsive element (ERE), and auxin-responsive element (TGA-element) (Figure 4). In addition, stress-stimulating elements such as anaerobic element (ARE), low temperature element (LTR), stress response element (STRE) and drought element (MSB), plant development-related elements, and myb and myc transcription binding sites exist in *LeBAHDs* promoters, which may possibly regulate their expression (Figure 4). Overall, certain elements such as Box 4, G-Box, ERE, and STRE were identified as high-frequency elements in the promoters of *LeBAHDs* (Figure S3). Importantly, the promoter region of the acyltransferase gene *LeBAHD1*/*LeSAT1* was enriched with cis-acting elements related to the light signal, ethylene, fungal initiators, etc., for the biosynthesis of shikonin and its derivatives (Figure 4).

### 3.5. Induction and Identification of the Transgenic Hairy Roots

**Figure 5:**
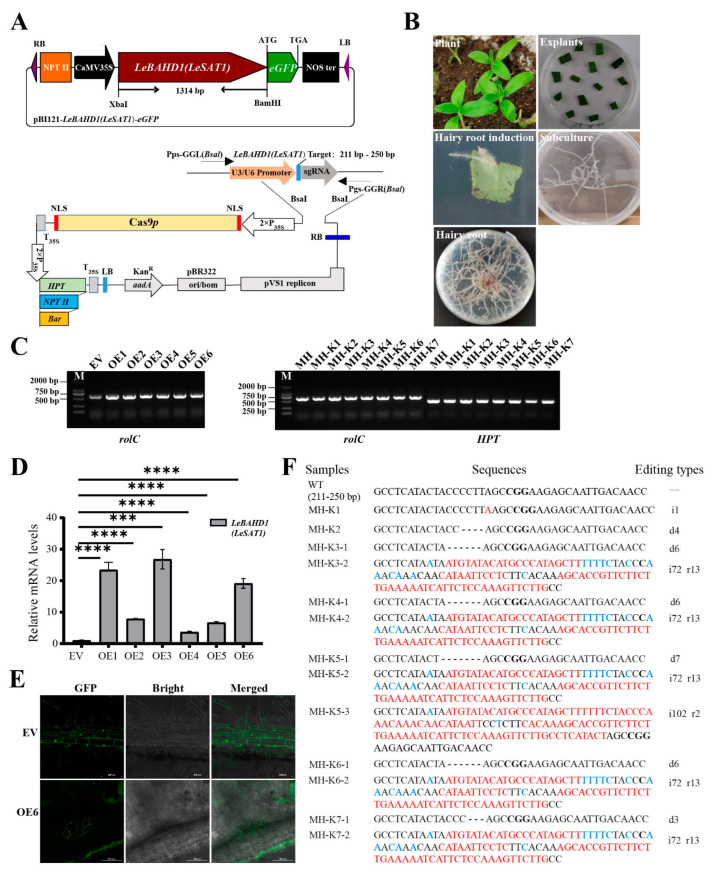
Generation and identification of transgenic hairy roots. (**A**) The structural maps of pBI121–*LeBAHD1*/*LeSAT1*–eGFP overexpression vector and pYLCRISPR/Cas9–*LeBAHD1*/*LeSAT1* knockout vectors. (**B**) Induction and subculture of EV, OE, MH and MH-K in *L. erythrorhizon* hairy roots. (**C**) PCR verification of the *rolC* gene in the *L. erythrorhizon* hairy root lines EV, OE1-6, MH, and MH-K1-7, and the *HPT* gene in MH, MH-K1-7. (**D**) Transcript levels of *LeBAHD1*/*LeSAT1* in the overexpression hairy roots cultured in 1/2 B5 multiplication medium under light at 26–28 °C. Asterisks indicate significant differences between each OE lines and EVs by the Student’s *t*-test. *** *p* < 0.001, **** *p* < 0.0001. All data are means ± SD (n = 3). (**E**) Subcellular localization of LeBAHD1/LeSAT1 in EV and OE6 *L. erythrorhizon* hairy roots. Scale bar = 100 μm. (**F**) Gene editing types of each of MH-K knockout hairy root lines, i: number of inserted bases, marked with red; d: number of missing bases, marked with a dash; r: number of replaced bases, marked with blue.

The function of *LeBAHD1/LeSAT1* in catalyzing the conversion of shikonin to acetylshikonin has been verified in vitro using the heterologously expressed protein in *E. coli*. In order to further confirm the function of *LeBAHD1/LeSAT1* in vivo in *L. erythrorhizon*, we constructed *A. rhizogenes* ATCC15834 strains transformed with overexpression and knockout (CRISPR-Cas9 system) plasmids of *LeBAHD1/LeSAT1* (Figure 5A), then a series of transgenic hairy root lines for EV (pBI121 empty vector), OE, MH (CRISPR/Cas9 empty vector), and MH-K were generated using the method we reported previously [34] (Figure 5B). Furthermore, the marker *rolC* gene of ATCC15834 and the marker *HPT* of CRISPR/Cas9 vector were amplified from the obtained series of EV, OE, MH, and MH-K hairy roots, confirming the effective acquisition of transgenic hairy roots (Figure 5C).

The results showed that the expression levels of *LeBAHD1/LeSAT1* were up-regulated to varied degrees in the six OE hairy root lines compared to EV, with the expression levels in the OE1, OE3 and OE6 lines being significantly raised by 23-, 27- and 19-fold, respectively (Figure 5D). As a result, these three lines were used as subsequent experimental materials. In addition, eGFP fluorescence detection was performed to identify the successful induction of EV and OE hairy roots as well as the subcellular localization of *LeBAHD1/LeSAT1*. In contrast to the apparent triple distribution of GFP in the PM, cytoplasmic space, and nucleus of EV hairy roots, GFP fluorescence was primarily found in the cytoplasm of OE hairy roots, which was consistent with the cytoplasm localization prediction of LeBAHD1/LeSAT1 (Figure 5E, Table S4), suggesting that the LeBAHD1–eGFP fusion protein was successfully expressed under the control of the 35S promoter.

Meanwhile, to investigate the impact of gene editing, *LeBAHD1/LeSAT1* sequences from the MH and MH-k hairy roots were amplified and sequenced. The findings verified the generation of knockout hairy root lines and the first effective use of the CRISPR-Cas9 system for gene editing in *L. erythrorhizon* hairy roots. The nucleotide sequences of *LeBAHD1/LeSAT1* in the seven knockout hairy root lines (MH-K1~MH-K7) were edited with nucleotide insertion, deletion, and/or substitution (Figure 5F). Among them, MH-K1 and MH-K2 were homozygotes, inserting one base and deleting four bases, respectively, in 211–250 bp of the *LeBAHD1/LeSAT1* coding sequence (CDS); MH-K5 contained three editing types in 211–250 bp of the *LeBAHD1/LeSAT1* CDS: (1) 7 bases are missing, (2) 72 bases are inserted and 13 bases are replaced, (3) 102 bases are inserted and 2 bases are replaced (Figure 5F). These three lines were then selected for the identification of acyltransferase function of LeBAHD1/LeSAT1 in *L. erythrorhizon*.

### 3.6. LeBAHD1/LeSAT1 Confers the Conversion of Shikonin to Acetylshikonin In Vivo in L. erythrorhizon Hairy Roots

**Figure 6:**
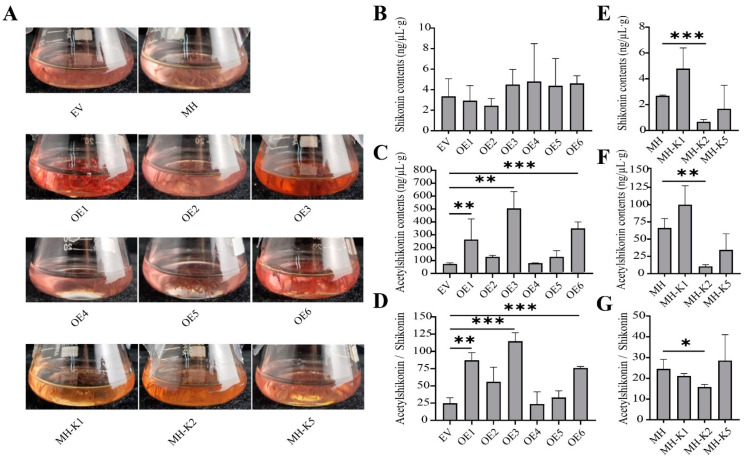
Color observation of the hairy root culture medium and HPLC analysis of shikonin and its derivatives in control (EV, or MH), overexpression (OE), knockout (MH-K) hairy roots of *L. erythrorhizon*. (**A**) Color observation of control (EV and MH) and transgenic hairy roots (OE and MH-K) cultured in M9 in darkness for 9 days. (**B**) Content analysis of shikonin in EV and OE hairy roots cultured in M9 in darkness for 9 days using HPLC analysis. (**C**) Content analysis of acetylshikonin in EV and OE hairy roots cultured in M9 in darkness for 9 days using HPLC analysis. (**D**) The ratio of acetylshikonin to shikonin in EV and OE hairy roots cultured in M9 in darkness for 9 days. (**E**) Content analysis of shikonin in MH and MH-K hairy roots cultured in M9 in darkness for 9 days using HPLC analysis. (**F**) Content analysis of acetylshikonin in MH and MH-K hairy roots cultured in M9 in darkness for 9 days using HPLC analysis. (**G**) The ratio of acetylshikonin to shikonin in MH and MH-K hairy roots cultured in M9 in darkness for 9 days. Asterisks indicate significant differences by Student’s *t*-test. * *p* < 0.05, ** *p* < 0.01, *** *p* < 0.001. All data are means ± SD (n = 3).

To investigate the role of LeBAHD1/LeSAT1 in enhancing acetylshikonin production in vivo in *L. erythrorhizon*, we measured the amount of shikonin and its derivatives produced by EV, MH, OE and MH-K hairy roots incubated in M9 medium for 9 days in the dark. Observing the color of the cultured transgenic hairy roots revealed that the color of three OE lines (OE1, OE3 and OE6) was significantly more red than that of the control EV and MH, whereas the color of the three knockout hairy root lines appeared yellow (Figure 6A). The pigment extracted from each hairy roots’ culture was then analyzed using HPLC. According to the peak diagram of each sample, the content of shikonin at 4.061 min retention time in all hairy roots was significantly lower than acetylshikonin at 4.792 retention time. The relative quantitative results showed that the content of shikonin in OE1, OE3, and OE6 was not significantly different from that of EV (Figure 6B); however, the amount of acetylshikonin produced from the three OE lines was significantly higher than that of EV by 3.58-, 3.92-, and 2.72-fold, respectively (Figure 6C). Statistically, the ratio of acetylshikonin to shikonin in the OE1, OE3, and OE6 was significantly increased by 3.5-, 4.6-, and 3.1-fold, respectively, when compared to the relative control (Figure 6D). Shikonin and acetylshikonin content, as well as the ratio of these two metabolites, was significantly lower in MH-K2 than in the control line MH, whereas there was no significant difference in pigment content or ratio in the other lines (Figure 6E–G).

## 4. Discussion

Traditional Chinese medicinal herbs, such as *L. erythrorhizon* and *A. euchroma*, get their significance from the secondary metabolites accumulating in their roots, primarily shikonin and its derivatives, which have a variety of pharmacological activities [35]. After paclitaxel and camptothecin, shikonin and its dervatives are regarded as promising natural antitumor agents due to their excellent anticancer activity [35]. Acetylshikonin is converted from shikonin by acyltransferase, and its content is 15 times higher than shikonin in the red roots of *L. erythrorhizon*. Moreover, acetylshikonin is less toxic to normal cells while also being anti-cancerous, and it has greater medicinal potential than shikonin [11,12]. Therefore, it is of importance to demonstrate how shikonin is converted to acetylshikonin and to increase the content of acetylshikonin in the callus cells or hairy roots of *Boraginaceae* plants. 

The plant BAHD gene family members have been proven to have acyltransferase activity and participate in the biosynthesis of flavonoids, anthocyanins, and other secondary metabolites [25]. The BAHD gene family members in genomes of *A. thaliana*, *Populus tomentosa*, *Oryza sativa*, *Vitis vinifera* and other species have also been fully identified, with 55, 100, 84 and 52 members, respectively [36]. However, no results have been reported about the genome-wide identification of *BAHDs* in *Boraginaceae* plants. In the present study, we made a successful identification and characterization of 73 BAHD family members in the genome of *L. erythrorhizon*, and performed functional analysis for these genes based on evolutionary status and tissue expression patterns. In particular, the positive regulatory effect of the candidate gene *LeSAT1* in catalyzing the conversion of shikonin to acetylshikonin was verified by a in vivo transgenic strategy.

The 73 LeBAHD family members we identified included the 54 AAT/SAT’s members previously identified by Tang from the *L. erythrorhizon* genome [28]. Tang’s research firstly used LeSAT1/LeAAT1 amino acid sequences as queries to blast the *L. erythrorhizon* genome, the initial sequence was obtained based on sequence homology, and 54 members were then screened out by removing the sequences with redundancy or lack of conserved functional domains or conserved motifs [28]. However, not all members of the BAHD acyltransferase gene family could be screened out from the *L. erythrorhizon* genome by only using AAT/SAT amino acid sequences as a query. In the present study, the query Pfam domain PF02458 was firstly used to search the genome, and the initial 104 sequences were obtained based on the presence in the sequences of characteristic domains. After that 73 members were screened out by removing the sequences with redundancy or lack of conserved functional domains or conserved motifs. Although all of the 54 family members in Tang’s study were represented by our 73 members, the purpose and gene family definitions of the two studies differed: Tang focused on an alkannin/shikonin O-acyltransferase gene family and its evolutionary history [28], while we focused on the BAHD acyltransferase family and its characterization and function.

A lot of evidence shows that the BAHD family members are involved in a variety of biological reactions: Dv3MAT in *D. variabilis* and Sc3MaT in *Pericullis cruenta* are responsible for the modification of anthocyanins [37,38], while NtMAT1 in *N. tabacum* is responsible for the modification of flavonoid and napthol glucosides [39]; based on the evolutionary relationship, 17 LeBAHDs were clustered into the clade I with these three proteins (Figure 2), implying they might have similar functions. LE22341.1 is a member in clade II and may have similar functions to Glossy2 in maize and CER2 in *Arabidopsis,* which mainly regulates cuticle wax extension and prevents pathogen invasion [26,40] (Figure 2); five LeBAHDs may be able to acylate a nitrogen to generate the corresponding amide, similar to ACT in clade IV [26,41] (Figure 2). The 26 LeBAHDs belonging to clade V are closely related to AtHCT in *Arabidopsis* and BanAAT in *M. sapientum*, which may be involved in the biosynthesis of volatile esters, hydroxyl cinnamyl quate/oxalate [26,42,43,44] (Figure 2). The substrate of most members of BAHD III subfamily is alcohols, and most of these enzymes use acetyl-CoA as the main acyl donor [26,45,46]. For example, *DAT* isolated from *C. roseus* can catalyze the acetylation of alcohol substance deacetylvindoline to produce an anti-cancer alkaloid drug—ventolin [24,26] (Figure 2). Since the catalytic process from shikonin to acetylshikonin is an acetylation modification reaction of alcohol hydroxyl, we speculate that LeBAHD1/LeSAT1 in Branch III could acetylate shikonin to acetylshikonin.

The tissue expression pattern of genes also could reflect their possible functions in plants. According to the transcriptome analysis of six tissues of *L. erythrorhizon* (Figure 3), LE22341.1, a member in clade II, is predominantly highly expressed in ML and might be responsible for cuticle wax extension in the leaves and stems of *L. erythrorhizon* in order to prevent water loss and microbial infestation. Twelve *LeBAHD* genes displayed higher transcription levels in flowers, which may be related to the synthesis of flower-specific metabolites. *LeBAHD1/LeSAT1* and *LeBAHD56*(LE25525.1) are mostly strongly expressed in MR and/or PD, the principle biosynthetic tissues of shikonin and acetylshikonin in *L. erythrorhizon*, suggesting that they may be involved in the acylation of shikonin.

Recently, Oshikiri et al. identified LeBAHD1/LeSAT1 using comparative transcriptome and proteomic analysis of *L. erythrorhizon*, and its function of catalyzing acetyl-CoA as acyl donors to generate acetylshikonin was verified by heterologous expression systems and in vitro enzyme activity [27]. However, heterologous in vitro experiments cannot fully imitate the complex biosynthetic and regulatory mechanisms of secondary metabolites in plants. It is therefore necessary to confirm the in vivo role of *LeBAHD1/LeSAT1* in *L. erythrorhizon*. As a result, the function of the *LeBAHD1/LeSAT1* enzyme in catalyzing the acetylation of shikonin was studied further in this study by constructing the overexpression and knockout lines of transgenic hairy roots of *LeBAHD1/LeSAT1*. The results showed that the yields of acetylshikonin, as well as the ratio of acetylshikonin to shikonin, were significantly higher in the overexpression lines OE1, OE3, and OE6, compared to the control lines, confirming that *LeBAHD1/LeSAT1* positively regulates shikonin conversion to acetylshikonin. However, only the MH-K2 line out of the three knockout hairy root lines had significantly lower shikonin yield, acetylshikonin yield, and the ratio of acetylshikonin to shikonin than the MH line. As a result, this finding is insufficient to establish the specific acyl transferase function of LeBAHD1/LeSAT1 in *L. erythrorhizon*. It is likely that other important genes with functional redundancy, such as *LeBAHD56* (LE25525.1), which clusters in the same clade as LeBAHD1/LeSAT1 with high sequence homology and that has greater transcript levels in roots where shikonin and its derivatives are biosynthesized, may account for the phenotypic variability in knockout hairy root lines. Therefore, more single-gene knockout hairy root lines of *LeBAHD1/LeSAT1* and *LeBAHD56* (LE25525.1) and double-gene knockout hairy root lines of these two genes are required to clarify their contribution ratio in regulating shikonin’s conversion to acetylshikonin in *L. erythrorhizon* using a CRISPR/Cas9-based knockout system. After demonstrating that *LeBAHD56* (LE25525.1) acetylates shikonin, we can generate double-gene overexpression hairy roots to determine if they can produce more acetylshikonin than single-gene overexpression hairy roots.

Due to its high efficiency of gene editing, CRISPR/Cas9-based knockout technology has been widely utilized to study gene function in numerous plant species, including the model plants *A. thaliana* [47] and *O. sativa* [48], woody plants such as *Populus* [49] and *Malus pumila* [50], and medicinal plants such as *Salvia miltiorrhiza* [51] and *Dendrobium officinale* [52]. However, previous studies have not reported the application of CRISPR/Cas9 technology in *Boraginaceae* plants. In this study, in addition to constructing *LeBAHD1/LeSAT1*-overexpressing transgenic hairy root lines to study their acyltransferase function, we also successfully introduced the CRISPR/Cas9 technology into the hairy root system of *L. erythrorhizon* and produced seven knockout lines with a total of seven gene-editing types, of which MH-K1 and MH-K2 were insertion homozygous and deletion homozygous, respectively, and MH-K5 contained three different editing types. This investigation of the practical feasibility of CRISPR/Cas9 technology in *L. erythrorhizon* can provide valuable research experience and methods for more precise characterization of the biosynthesis and regulatory pathways of shikonin and its derivatives.

In conclusion, our study employed a successful identification and characterization of *LeBAHD* family members from *L. erythrorhizon.* We confirmed *LeBAHD1/LeSAT1*’s function in the biosynthesis of acetylshikonin by converting shikonin in the transgenic hairy root system in vivo utilizing overexpression and CRISPR/Cas9-based knockout transgenic experiments. We also provided some potential candidate genes with functional redundancies to *LeBAHD1/LeSAT1* in the same phylogenetical branch with similar expression patterns, such as *LeBAHD56*(LE25525.1). Our findings not only contribute to a better understanding of the regulatory mechanism governing the biosynthesis of shikonin and its derivatives in *L. erythrorhizon* or other secondary metabolites in non-model medicinal plants, but also offer compelling evidence that there is a possibility to produce high yields of acetylshikonin in in vivo in *L. erythrorhizon* by manipulating *LeBAHD1/LeSAT1* through genetic engineering.

## Data Availability

All supporting data can be found within the manuscript and its supplementary files.

## Supplementary Materials

The following supporting information can be downloaded at: https://www.mdpi.com/article/10.3390/life12111775/s1, Figure S1. Logos of 15 conserved motifs were identified in 73 LeBAHDs from *L. erythrorhizon* using the MEME search tool. Figure S2. The expression level of *LeBAHD1/LeSAT1* in the leaf, stem, and root of *L. erythrorhizon* seedlings were analyzed using RT-qPCR. Figure S3. Cloud word graph of cis-acting elements in *LeBAHDs*’ promoters. Table S1. Basic information about published BAHD reference genes in D’Auria’s study. Table S2. Primers used for experiment of *LeBAHD1/LeSAT1* on *L. erythrorhizon*. Table S3. Identification of BAHDs from the genome of *L. erythrorhizon*. Table S4. The basic information of *LeBAHDs* in *L. erythrorhizon.* Table S5. The number of cis-acting elements in *LeBAHDs*’ promoters.
