# Supplementary material for: Genome-Wide Identification of LeBAHDs in Lithospermum erythrorhizon and In Vivo Transgenic Studies Confirm the Critical Roles of LeBAHD1/LeSAT1 in the Conversion of Shikonin to Acetylshikonin
Source: Life (Basel). 2022 Nov 3;12(11):1775. doi: 10.3390/life12111775 (PMC9694994; doi:10.3390/life12111775)

**Figure S1** Logos of 15 conserved motifs were identified in 73 LeBAHDs from *L. erythrorhizon* using the MEME search tool.

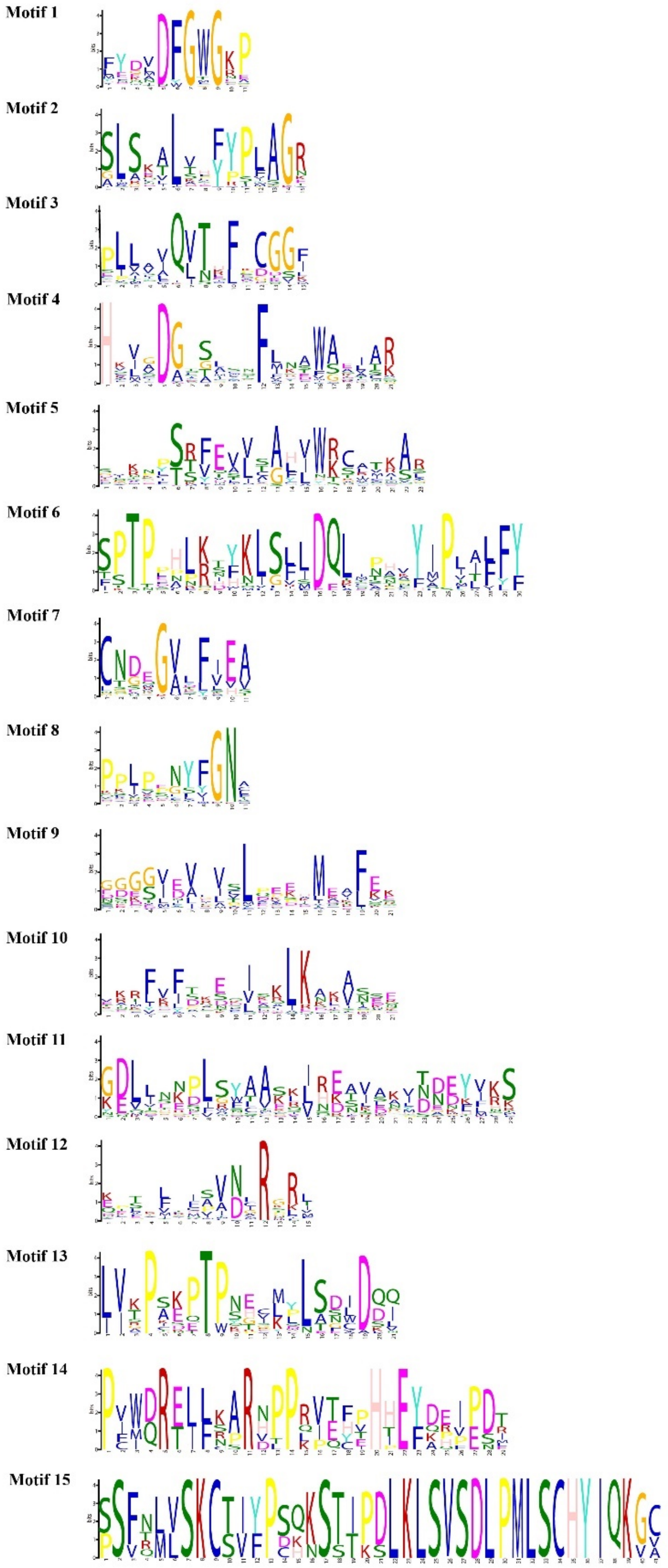

Supplement: Supplementary file 1 [file life-12-01775-s001.zip › life-1953751-R2-Supplemental data-FIN/Figure S1_R2.pdf]
