# Supplementary material for: Genome-Wide Identification of LeBAHDs in Lithospermum erythrorhizon and In Vivo Transgenic Studies Confirm the Critical Roles of LeBAHD1/LeSAT1 in the Conversion of Shikonin to Acetylshikonin
Source: Life (Basel). 2022 Nov 3;12(11):1775. doi: 10.3390/life12111775 (PMC9694994; doi:10.3390/life12111775)

Figure S2 The expression level of *LeBAHD1/LeSAT1* in the leaf, stem, and root of *L. erythrorhizon* seedlings were analyzed using RT-qPCR. Asterisks indicate significant differences between each root, steam and leaf by Student's t-test. $**p < 0.01$. All data are means ± SD (n = 3).

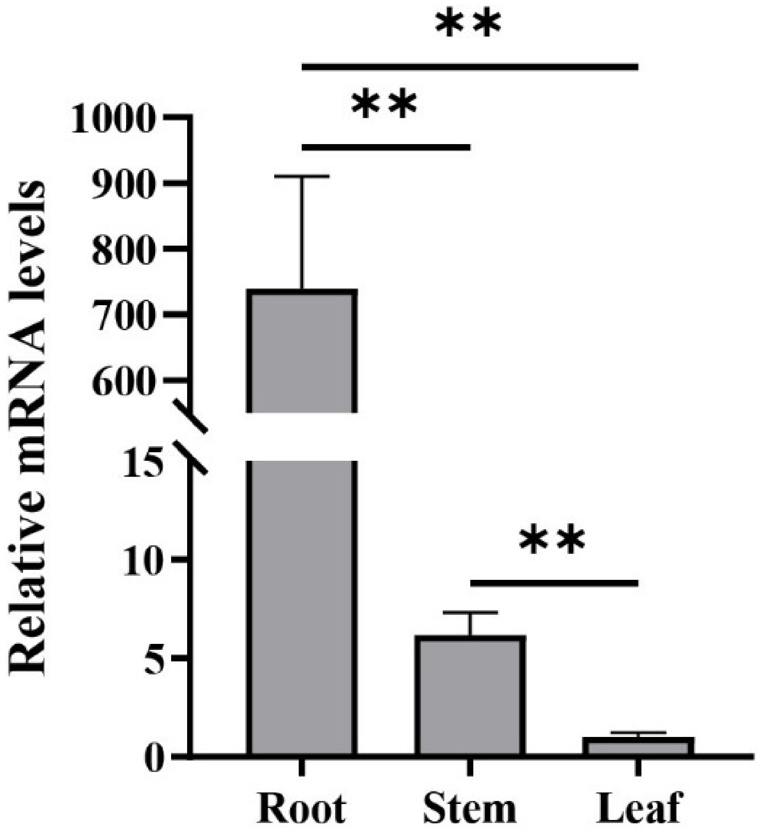

Supplement: Supplementary file 1 [file life-12-01775-s001.zip › life-1953751-R2-Supplemental data-FIN/Figure S2_R2.pdf]
